# Geopolitics and public health: Europe under the shadow of the U.S. National Security Strategy

**DOI:** 10.1016/j.lanepe.2026.101625

**Published:** 2026-02-12

**Authors:** Jonathan Cylus, Martin McKee

**Affiliations:** a European Observatory on Health Systems & Policies, London School of Hygiene & Tropical Medicine, 15-17 Tavistock Place, London, WC1H 9SH, United Kingdom; b Department of Health Services Research and Policy, London School of Hygiene & Tropical Medicine, 15-17 Tavistock Place, London, WC1H 9SH, United Kingdom

**Keywords:** Security, Europe, Populism, United States

## Abstract

The 2025 U.S. National Security Strategy marks a major shift toward a narrowly defined “America First” agenda with significant implications for Europe and its health systems. It reframes global public goods, including disease surveillance, migration governance, and climate action, as burdens rather than shared responsibilities, while prioritising hard power, economic nationalism, strict border control, and fossil fuel expansion. Europe is urged to increase defence spending to 5% of GDP and reduce regulatory and welfare commitments, thereby creating fiscal pressures that risk crowding out investment in health and social care. Its rhetoric on migration, culture, and declining birth rates aligns with and empowers far-right movements, potentially accelerating welfare retrenchment and undermining access to care for migrant and marginalised populations. This Viewpoint analyses the implications of the 2025 U.S. National Security Strategy for public health and health systems in Europe, with particular attention to welfare, migration, climate, and multilateral cooperation. A weakened WHO, reduced U.S. multilateral engagement, and more transactional transatlantic relations threaten global health security. Europe must safeguard health systems, equity, and the multilateral global order.

## Introduction

The 2025 United States (U.S.) National Security Strategy (NSS) marks a decisive departure from its predecessors. It shifts away from global engagement and the provision of global public goods toward a narrow ‘America First’ agenda that redefines national interest, elevates sovereignty, and subordinates multilateralism to domestic political, economic, and cultural priorities.^1^

The NSS, although framed as a foreign and security policy document, has broader implications. It reflects a growing ideological project in U.S. politics that blends sovereigntist themes with nativist and cultural politics. Seeing demographic change as a security threat, the ‘America First’ agenda—and much of the NSS—emphasizes tightening borders, resisting supranational regulation, and reframing global public goods as burdens. The health implications are clear. If implemented, the NSS could reshape global political and economic systems that underpin population health, ranging from disease surveillance and migration to trade, energy, and economic governance.

The NSS frames these domains primarily as national security imperatives for the United States to determine rather than as shared responsibilities to be collectively agreed upon. This has profound implications for Europe, where health systems are deeply embedded in welfare states, regulatory regimes, and supranational institutions that the NSS portrays as obstacles to U.S. power. Understanding what this strategy means for health, therefore, requires asking three questions. What objectives are the U.S. pursuing in the NSS? What does the U.S. want Europe to do to enable it to achieve them? And what might European responses mean for the continent's health systems and the broader determinants of health?

## What does the U.S. want?

The objectives of the 2025 NSS are, in many ways, a continuation of earlier ones, prioritising U.S. security, economic strength, and geopolitical influence in an uncertain world. However, the ways these objectives are pursued and the assumptions underpinning them represent a significant break with U.S. strategy since World War II.

The NSS redefines security in narrowly national terms. Rather than viewing international cooperation and strong multilateral institutions as means to achieve global stability and as security assets, it treats them as contingent and expendable. Thus, “*The United States will unapologetically protect our own sovereignty. This includes preventing its erosion by transnational and international organizations”* (p10) and “*We stand for the sovereign rights of nations, against the sovereignty-sapping incursions of the most intrusive transnational organizations …”* (p9), although its appeal to sovereignty is selective, invoked robustly where it aligns with U.S. objectives and downplayed where it does not (for example, in positions on Ukraine, Venezuela, and Greenland). Global public goods, such as pandemic preparedness, climate mitigation, international disease surveillance, development assistance, and humanitarian response, are framed not as collective investments that ultimately protect the U.S., but as burdens that dilute sovereignty and divert resources from domestic priorities. This is especially so for development assistance, health systems strengthening, and humanitarian response, long seen as benefitting all nations and not just those directly affected. The NSS's re-prioritisation of domestic objectives and the restructuring of U.S. development institutions since 2025 signal a broader retreat from these commitments.

The withdrawal from the World Health Organization^2^ was an early signal of this broader shift that deepened through 2025. Global health security, once understood as indivisible, is reimagined as a discretionary activity, justified only insofar as it produces immediate and tangible benefits for the U.S. Second, the NSS places economic power at the centre of national security thus: “*Because economic security is fundamental to national security, we will work to further strengthen the American economy …”* p13. Economic growth is framed not as a social objective but as a strategic necessity, tied to industrial capacity, technological leadership, energy dominance, and military readiness. The document repeatedly highlights Europe's relative economic decline thus: “*Continental Europe has been losing share of global GDP—down from 25% in 1990 to 14% today … The larger issues … include … transnational bodies that undermine sovereignty, migration policies … cratering birthrates, and loss of national identities …”* p25. Weak growth is presented as both a symptom and a cause of strategic vulnerability. Regulatory intensity, welfare commitments, and acceptance of supranational governance, in this case the European Union (EU), are portrayed as impediments to competitiveness, innovation, and national renewal.

Throughout the document, the NSS obsesses over cultural cohesion. It frames demographic change resulting from the effects of migration and declining birth rates as existential threats to national identity and stability, particularly in Europe and the U.S. Migration, in particular, is securitised in language that echoes the logic of the Great Replacement Theory, portraying population movement not as a public good and an economic necessity in destination countries in light of population ageing and shrinking workforces but as a threat to civilisation itself.^3^ It talks of “*the real and more stark prospect of civilizational erasure*” (p25)*.* Strict border control is elevated from a policy choice to a defining feature of sovereignty, stating that “*Who a country admits into its borders … will inevitably define the future of that nation … The era of mass migration must end. Border security is the primary element of national security*” (p11).

The strategy also emphasises hard power and burden-sharing. NATO allies are urged to commit 5% of GDP to defence, a dramatic increase from existing targets. While deterrence and strategic stability are important to Europe, the NSS demand reflects a broader U.S. objective: to reduce its relative contribution to European security while compelling allies to align more closely with U.S. priorities.

Finally, the NSS rejects climate mitigation agendas, including “Net Zero,” in favour of energy dominance and the expansion of fossil fuels, condemning Europe specifically: “*We reject the disastrous ‘climate change’ and ‘Net Zero’ ideologies that have so greatly harmed Europe …*” p14. Control over critical minerals, energy reserves, and industrial inputs is framed as central to geopolitical power. This resource-first worldview places strategic assets above environmental protection and population well-being.

Taken together, these objectives reveal a transactional vision of security: national power derives from economic strength, cultural cohesion, territorial control, and military capacity, rather than from international cooperation or social investment. Health is subsumed within this logic rather than treated as a strategic domain in its own right.

The NSS's framing is consistent with the America First Global Health Strategy, which subordinates global health engagement to domestic strategic returns. Together, these documents normalise conditionality in partnerships, prioritise bilateral over multilateral modalities, and risk fragmenting global-health coordination, outcomes that would shift burdens onto European actors if left unaddressed."

## What is the U.S. expecting Europe to do?

The NSS does not examine threats to Europe or articulate how the United States might help address them. Instead, it is preoccupied with what Europe should do to improve its standing in Washington's view. The strategy's implications for Europe are therefore best understood not as protective but as directive.

First, the U.S. wants Europe to assume greater responsibility for its own security, and on U.S. terms. The call for 5% defence spending has implications beyond military capability; it forces a reorientation of fiscal and political priorities. By forcing European governments to prioritise defence within constrained budgets, the strategy implicitly encourages trade-offs that weaken welfare states and social investment. These pressures are likely to be particularly acute in countries already experiencing slow growth and fiscal stress, including Germany, France, and the United Kingdom.

Second, the U.S. seeks to create economic conditions favourable to U.S. interests in Europe. This includes reducing regulatory barriers to trade, liberalising markets, and aligning industrial and trade policies more closely with U.S. priorities. While the NSS does not explicitly mention the regulation of health care markets, the document's logic suggests that policies perceived as constraining U.S. exporters, such as stringent pharmaceutical pricing policies or health technology assessment thresholds, may be targeted. In England, ministers seem to have accepted a rise in the reference cost-effectiveness threshold used by NICE from £25,000 to £35,000 per QALY as a concession to obtain tariff-free UK pharmaceutical exports to the U.S.,^4^ agreed to even though evidence already shows that drugs approved at the previous threshold crowd out spending on health services that could have provided better value for money.^5^

Above all, the NSS appears designed to resonate with and empower right-wing and populist political movements in Europe. Its language on migration, welfare dependency, and cultural decline closely mirrors the narratives of parties that have long sought to restrict immigration, roll back welfare provision, and weaken supranational governance.^6^ While it would be simplistic to claim that the U.S. actively seeks to engineer electoral outcomes in the way that Russia does,^7^ the strategy's framing clearly legitimises political forces that align with its worldview. By amplifying fear-based narratives around migration and identity, the NSS contributes to a political climate where retrenchment of social protection becomes more politically feasible. It could also bolster support for populist politicians by creating the impression that they would be treated more favourably by the U.S. if elected, as appears to have happened in Argentina in 2025.^8^

It is clear that the U.S. wants a weaker and more fragmented Europe. The NSS repeatedly criticises supranational governance, particularly the EU, which it frames as an obstacle to sovereignty and economic dynamism. The NSS condemns it thus: the “*European Union and other transnational bodies that undermine political liberty and sovereignty, migration policies that are transforming the continent and creating strife, censorship of free speech and suppression of political opposition, cratering birthrates, and loss of national identities and self-confidence*” p25. The reference to ‘free speech’ also signals pushback against EU efforts to regulate large U.S. technology and social-media platforms, on taxation, competition, data, and online harms.

A Europe composed of more inward-looking, nationally oriented states is easier to engage transactionally, whether on defence, trade, or energy. Here, it may be having some success, with differences emerging among some EU member states on how to deal with the U.S. (although President Trump's threatening rhetoric and behaviour towards Europe, not least with respect to Greenland, may serve to unite them). This logic of divide-and-rule extends to international organisations more broadly. Reduced U.S. engagement with bodies such as the WHO signals that multilateralism is no longer a cornerstone of U.S. strategy, encouraging others to follow suit or at least to deprioritise collective action.

In short, the NSS envisages a Europe that is more militarised, more economically liberal, more fragmented across borders, and more politically aligned with U.S. interests.

## What might this mean for health and health systems in Europe?

Does the U.S. vision of Europe come at the expense of its welfare states? We believe the health implications of the U.S.'s objectives are profound, even if they come about indirectly through the political, economic, and institutional choices that European governments may feel compelled or empowered to make.

The demand for 5% defence spending represents a significant opportunity cost. Because health and social care constitute large, recurrent portions of national budgets, increases in defence spending within fixed fiscal rules almost inevitably force governments to reduce growth in health budgets, delay capital investment, or restrict workforce expansion. Health systems are, however, as critical to societal resilience as military capacity, as demonstrated during the Covid pandemic. Yet they lack the symbolic and political appeal of defence spending in a securitised environment. The likely political response to these pressures is not increased taxation but welfare retrenchment, particularly in contexts of slow economic growth and shrinking labour markets due to population ageing. Health systems, already struggling with workforce shortages, the care needs of ageing populations, and rising costs, will struggle to do more with less.

If far-right and populist parties gain influence, as the NSS's framing may encourage, health systems may be threatened by deliberate policy choices rather than fiscal necessity alone. Such parties often question the legitimacy of redistributive welfare states, particularly where beneficiaries are portrayed as outsiders or undeserving. Migrant populations may face restricted access to healthcare, reduced preventive services, and increased exposure to discrimination, with consequences not only for their own health but for population health more broadly. Sexual and reproductive rights, still limited in some parts of Europe, risk attack.

Stricter migration controls may also exacerbate workforce shortages in health and social care, particularly in nursing and elder care, where reliance on migrant labour is high.^9^ Reduced mobility of health professionals undermines national resilience. Recent U.S. experience shows how securitised narratives about migration can harden into policy. Eligibility rules, documentation hurdles, and targeted exclusions have reduced access to primary care, public insurance, and social assistance for migrants and mixed-status families, with spillovers to public health. If adopted in Europe, such approaches would likely widen coverage gaps, deter timely care-seeking, and heighten avoidable morbidity while deepening social division.

A more transactional transatlantic relationship also increases the risk that health policy becomes subordinated to trade and industrial objectives, something already happening within the European Commission's Health Directorate General.^8^ Pressure to relax pharmaceutical pricing controls, fast-track market access, or weaken regulatory standards may be justified in the name of competitiveness and support for industry, but it could have negative health consequences through increased costs borne by health systems already under pressure or premature approval of novel drugs without full appreciation of their risks.

The NSS's rejection of climate mitigation and prioritisation of fossil fuel expansion may exacerbate health risks associated with air pollution and climate change by increasing exposure to extreme heat, flooding, and wildfires, and by worsening respiratory and cardiovascular diseases linked to air pollution.^10^ These environmental stresses will disproportionately affect vulnerable populations, strain health systems already under fiscal pressure, and undermine progress on climate-related health adaptation. In addition, Europe's ability to meet its own climate and health targets may be compromised if transatlantic trade and energy policies incentivise fossil-fuel dependence rather than a green transition.

Finally, reduced commitment to international organisations undermines collective capacity to detect and respond to health threats. Global disease surveillance, vaccination coordination, and pandemic preparedness depend on trust, data sharing, and sustained investment. A weakened WHO and stalled pandemic agreement increase vulnerability to future outbreaks, with Europe unlikely to be insulated from their effects.

## Conclusion

The 2025 U.S. NSS reshapes transatlantic relations in ways that will inevitably affect health and health systems in Europe, not because it targets health directly but because it reframes the political and economic structures on which health depends. Its overriding emphasis on sovereignty, national economic power, migration control, and rapid militarisation encourages European governments to make fiscal and regulatory choices that may weaken welfare states, crowd out investment in health and social care, and sideline collective approaches to health security. Although many consequences would emerge indirectly through the incentives the NSS creates, some are consistent with and may reflect explicit intent.

Europe need not treat the NSS as a fixed constraint. More robust commitments to protecting fiscal space for health, maintaining stringent health-technology assessment standards, safeguarding migrant access to care, and investing in climate mitigation counteract the dynamics the NSS sets in motion. Diplomatic strategy should prioritise coalition-building with U.S. partners in Congress, States, cities, universities, and civil society who share an interest in robust transatlantic cooperation on health, climate, and science. European institutions can table coordinated positions at the UN and other fora to defend multilateral health governance and migrant rights. When warranted, the EU and willing states should leverage their market power, through procurement, standard setting, and access to investment, to secure guardrails around health, data, and environmental protections in transatlantic engagements.

The challenge for Europe, therefore, is not only to navigate a shifting security environment but to prevent that shift from eroding the social, institutional, and political foundations of health. This requires distinguishing among cases in which alignment with U.S. objectives is strategically necessary, optional, or unacceptable due to the health consequences. Protecting health systems, upholding equity, and sustaining the cooperative frameworks essential for health security should remain non-negotiable elements of Europe's response.

## Contributors

Both authors contributed equally.

## Use of AI

Microsoft Copilot was used to undertake an initial search of the text of the NSS for quotes, all of which were checked manually.

## Declaration of interests

None declared.
